# The Perception of Physical Therapy Students towards Their Profession in Jordan

**DOI:** 10.3390/healthcare10050849

**Published:** 2022-05-05

**Authors:** Mikhled F. Maayah, Muhammed Al-Jarrah, Sunitha Mysore, Riziq Allah Gaowgzeh, Umar M. Alabasi, Thamer A. Altaim, Ziyad Neamatallah, Saad S. Alfawaz

**Affiliations:** 1 Department of Rehabilitation Sciences, Faculty of Applied Medical Sciences, Jordan University of Science and Technology, Irbid 22110, Jordan; jarrahm@just.edu.jo or; 2 Department of Occupational Therapy, Faculty of Medical Rehabilitation Sciences, King Abdulaziz University, Jeddah 21589, Saudi Arabia; 3 Department of Physiotherapy, Fatima College of Health Sciences, Abu Dhabi 3798, United Arab Emirates; sunitha.mysore@fchs.ac.ae; 4 Department of Physical Therapy, Faculty of Medical Rehabilitation Sciences, King Abdulaziz University, Jeddah 21589, Saudi Arabia; rizikjoresearch@gmail.com (R.A.G.); ualabasi@kau.edu.sa (U.M.A.); zneamatallah@kau.edu.sa (Z.N.); saalfawaz@kau.edu.sa (S.S.A.); 5 Physical Therapy Division, Allied Medical Sciences Department, Aqaba University of Technology, Aqaba 11191, Jordan; thamerpt@live.co.uk

**Keywords:** perceptions, physical therapy program, profession

## Abstract

Background: The physical therapy profession has grown rapidly in less than a century, increasing its importance, techniques, settings, and the responsibilities provided to its practitioners. **Objectives:** The aim of the study was to explore how undergraduate physiotherapy students view physiotherapy as their future career and their perception of the educational physiotherapy programs in Jordan. Methods: A cross-sectional study was conducted. A questionnaire designed to collect data on students’ perceptions of their profession was delivered to 222 undergraduate physiotherapy students at Jordan University of Science and Technology in Irbid, Jordan. The response rate was 157 (70.72%). Descriptive statistics and the chi-square test were used to analyse the data. Results: Among 157 physiotherapy students, results were collected. Although students were satisfied with being physiotherapy students (*p* < 0.001), most of the study participants knew about physical therapy from their families, and they were not satisfied with the job opportunities in Jordan. Conclusions: Physiotherapy education in Jordan is rising rapidly. The public, patients, parents, and clinical preceptors in physiotherapy settings must recognize this educational and professional practice. It is noted that some levels of occupational awareness are intermediate. However, it would be beneficial to organize activities such as seminars and interviews in order to increase the level of professional awareness.

## 1. Introduction

The profession of physical therapy has grown rapidly in less than a century, increasing its roles, procedures, and settings and the responsibilities provided to its practitioners by adjusting to changing demographics, social, and political circumstances [1,2,3]. Furthermore, the situation and profile of a profession are very dependent on the common interests of its members [2]. The development of a professional identity is based on the process of professional socialization that requires individuals to adopt rules, beliefs, and standards recognized by others who are or may become members [4].The theory of professional socialization emphasizes students’ engagement with the target area, their preferences and choices, and their role models [4].The achievement of educational outcomes for professions through student socialization is possible through a profession’s values, attitudes, and beliefs and commitment to a career [5].

The field of physiotherapy is a dynamic profession that offers tremendous opportunities in terms of academic learning, clinical practice, and research. Today, physiotherapists are autonomous professionals who take active roles in the prevention of disease and the promotion of wellness as well as physical fitness and rehabilitation [6]. Many comprehensive studies on perceptions of the profession of physiotherapy as well as its involvement in healthcare have been conducted globally. These investigations elicited data from various groups, including high school students [7], future physiotherapists [2], other medical practitioners [8,9], and practicing physiotherapists [9]. Many studies have been conducted to compare the profession to other occupations [2,9].

Physical therapy is a healthcare profession that involves evaluation and diagnosis to cure impairments, improve mobility, and hence improve quality of life. Therefore, the academic bachelor’s degree programs in three universities in Jordan adopt the principles of professional physiotherapy practice by equipping the students with distinguished scientific and clinical skills that include hands-on training in all physiotherapy clinical settings. In Jordan, the factors that influence students to enter a discipline within the allied health sciences are not well understood. Unlike in the UK or Australia, students in Jordan need not demonstrate their understanding of the profession and gain clinical experience before entering a healthcare program. The student selects a healthcare program based on his or her cumulative average in high school by standardized admission to the kingdom.

Physical therapists need to provide a clear understanding of the meaning and aim of their profession and a deliberate knowledge of a professional identity that involves specific career goal interventions in evolving contexts throughout their career [10]. One gains knowledge of one’s career’s concepts, beliefs, and attitudes through the process of professional socialization [10], and one builds a commitment to professional life through the process of professional socialization. Over the past years, there has been growing interest in physical therapist specialists identified as professionals [11].

Physical therapy is a branch of applied medical sciences based on its knowledge, educational methods, and practical application, which is an important task for society [12]. Physical therapy provides the right assessment, diagnosis, planning, intervention, and evaluation of the patient’s treatment to the right client at the right time in addition to an increase in techniques [13]. Considering the range of professional activities, supervising clinical practice is essential for education and research [14]. The goals of physiotherapy treatment are typically to relieve pain, maintain or improve mobility, minimize disability, reduce stiffness and swelling, increase range of motion and strength, maximize joint protection, and reduce stress in order to help the body return to its neutral state [15,16].

Physical therapy units offer medical services to all ages and carry out different activities, and most of the clients are working-aged people suffering from musculoskeletal conditions [17]. They are also responsible for the provision and maintenance of medical aids by the physiotherapist.

Physical therapists work and practice in many settings, such as governmental and non-governmental health settings, home settings, private-owned physical therapy clinics, outpatient clinics, health and wellness clinics, rehabilitation hospitals, extended care facilities, education, and research centers, fitness centers, and sports training facilities [2].

A small amount of research on the understanding of physical therapy among patients and non-patients, medical professionals, and students studying the field has been conducted in recent years [2]. The interest of physical therapy students in defining their professional identity has increased in recent years [18]. Education in physiotherapy over the past few decades and throughout the world has undergone a series of changes [19]. The most significant of these is the transfer of programs from colleges and hospitals to universities, where they have assumed applied sciences and clinical sciences frameworks [19]. Physical therapists have been educating themselves to improve their clinical skills, knowledge, and expertise and to have professional status, financial support, and legislative protection of their view of the body that distinguishes them from other practitioners [19]. The public needs to understand both the educational and professional changes that occur in physiotherapy to benefit the healthcare process. With the increase of physiotherapist independence, the public has “much to talk about whom, when and why health care is obtained from” [20]. The ability to understand public perceptions and knowledge of physiotherapy can help the profession in developing more successful methods and determining what can be done. It is also very important to advertise the perceived value of the profession to attract enough clinicians of the highest degree [2]. The perception of other careers, such as nursing and engineering, is different [21].

Physical therapy is a well-known healthcare profession in Jordan. However, the public’s knowledge of the wider scope of physiotherapy practice and post-graduate opportunities appears to be highly limited. Students entering the physiotherapy program in Jordan are rapidly increasing, and within a few years, there will be a high number of physiotherapists in the country. Student experience and satisfaction are highly important for any physiotherapy program, and to guide students effectively in their career, it is important to understand the current knowledge about the profession the student holds.

Faculty students have a wide variety of perspectives and ideas to select from when selecting a career path. In Jordan, high school students are not instructed about their future careers. Moreover, the graduates of academic institutions just recently received formal information about the needs of marketing through the governmental office of labor. It is also not clear to what extent the students who have enrolled in physiotherapy are aware of the scope of practice and future opportunities available to them after they graduate. Universities play a vital role in training students for future market demands, and physiotherapy, as a career with an increased scope of practice, has expanded. Hence, understanding a student’s awareness of the profession and exploring their future aspirations is paramount to both professionals and educational institutions. In 1999, three governmental universities in Jordan established a four-year degree in physical therapy to replace the two-year diploma. To this end, there is no study investigating the perception of physical therapy students regarding their profession in Jordan. Therefore, the main aim of this study was to explore how undergraduate physiotherapy students view physiotherapy as their future career and their perception of the educational physiotherapy programs in Jordan.

## 2. Materials and Methods

### 2.1. Study Design

A cross-sectional, questionnaire-based study was conducted in the faculty of applied medical sciences, department of rehabilitation sciences, physiotherapy division at Jordan University of Science and Technology (JUST), Irbid, Jordan, to evaluate physiotherapy students’ perceptions of their profession.

### 2.2. Participants

All students studying in the undergraduate physiotherapy program at JUST University (222) were invited to complete a survey in their first through fourth years. One hundred and fifty-seven (157) people between 18 and 28 years of age responded to the survey, of which 68 (43.31%) were female, and 89 (56.69%) were male. In addition, students from different nationalities are currently studying for a bachelor’s degree in physiotherapy at Jordan JUST. Of these, 33 were first-year students, 58 were second-year students, 47 were third-year students, and 19 were fourth-year students.

The number of students at the JUST university was about 222 students. According to the Yamane formula, the minimum required sample size was 141 students to obtain enough completed responses: *n* = N/(1 + N^2^), where *n* is the sample size, N is the population size, and = adjusted margin of error with a 95% confidence level [21].

### 2.3. Questionnaire

A survey questionnaire was modified based on the research needs and after consulting some of the literature on this topic. The questions included in the survey were mostly closed-ended with a predefined list of answer options except for question 12, which had an open-ended format. Various formats of survey questions were used, including the Likert scale, dichotomous (Yes/No) questions, checkbox questions, and a dropdown with options for both multiple and single answers. A variety of formats were used to capture as much information as possible in a simple and meaningful way. The questionnaire had two parts: Part A consisted of questions related to socio-demographic data regarding the gender, age, nationality, and year of study (levels).

Part B was to explore the student’s current awareness and perception of physiotherapy as a profession in Jordan. This section consisted of 13 questions covering five different dimensions: (1) factors influencing their entry into the physiotherapy program, (2) participants’ understanding of services offered to the patients, (3) their perception of the status of physiotherapy as a profession, (4) current problems faced by students, and (5) students’ satisfaction level in the program.

The information was gathered at the Jordan University of Science and Technology (JUST) in Irbid, Jordan. Data collection was started after getting approval from the JUST’s Institutional Research Board (approval number is 208/2016). A signed consent form was obtained from each student before study participation. There is not any type of hazard from participation in this study. A self-administered questionnaire with a validity of 0.83 was validated by five clinical experts for data collection. A team of five research experts verified this modified survey questionnaire by computing its content validity index and assessing the questions on clarity, simplicity, and relevance using a four-point Likert scale. Based on the pilot study, the questionnaire had a validity of 0.83 and a reliability of 0.728, and the overall reliability was 0.703.

### 2.4. Duration of Data Collection: Conducted over a Period of Five Months between 22 December 2017 until 24 April 2018

The duration of collecting data was during the second semester of the academic year between 22 December 2017 and 24 April 2018. Each participant provided a particular time to respond. It took about 15–20 min for each student to complete the questionnaire.

### 2.5. The Procedure of Data Collection

The questionnaire was distributed to undergraduate students in the fields of physiotherapy 222 (157 respondents). The questionnaire and demographic characteristics were administered to both students after a lecture class during the second semester of the academic year and collected with the aid of teaching assistants. They addressed the class before questionnaire administration regarding the purpose and process of data collection. It was also explained that they would use the data for the process of quality assurance, and it sought study objectives. The information was gathered at the JUST in Irbid, Jordan. Data collection was started after getting approval from JUST’s Institutional Research Board (approval number is 208/2016). A signed consent form was obtained from each student before study participation. The letter identified the researchers, the title, and the aim of the study. The code number for each student was maintained, and participation is voluntary.

### 2.6. Statistical Analysis

Statistical analysis of the study was conducted using IBM SPSS Statistics software for Windows (Version 22.0, IBM Corp. Armonk, New York, NY, USA). The descriptive statistics of correct answers to the questionnaire are mean ± standard deviation, given as frequency and percentage. A chi-square test was used to compare those who preferred the department with those who did not want it. The probability of error was accepted as *p* < 0.05 [21].

## 3. Results

A total of 157 (70.72%) participants, with mean-aged physiotherapy students (20.41 1.69) were involved in the study. There was female 89 (56.7%) and male 68 (43.3%) participants. The largest age group of the study participants was between the ages of 19 and 20 years, with a percentage of 26.4%, and the lowest age was between the ages of 25, 26, and 28 years, with a percentage of 12%. Most of the study participants were female 89 (56.7%), while the percentage of males was 68 (43.3%) from JUST. Most of the study participants by level were from the second year, with a percentage of 36.9%, and the lowest level was from the fourth year, with a percentage of 12.1%.

The demographic characteristics of the samples (*n* = 157) are shown in Table 1.

In Table 1, Jordanians comprise most of the study participants (82.2%), with Americans, Germans, Israelis, and Syrians accounting for the lowest percentage (0.6%). Furthermore, most of the study participants answered the question “Where did you learn about physiotherapy?” with the category of “family”, with a percentage of 30.6%, and the lowest category, with a percentage of 8.3%, was from “social media.”

**Figure 1:**
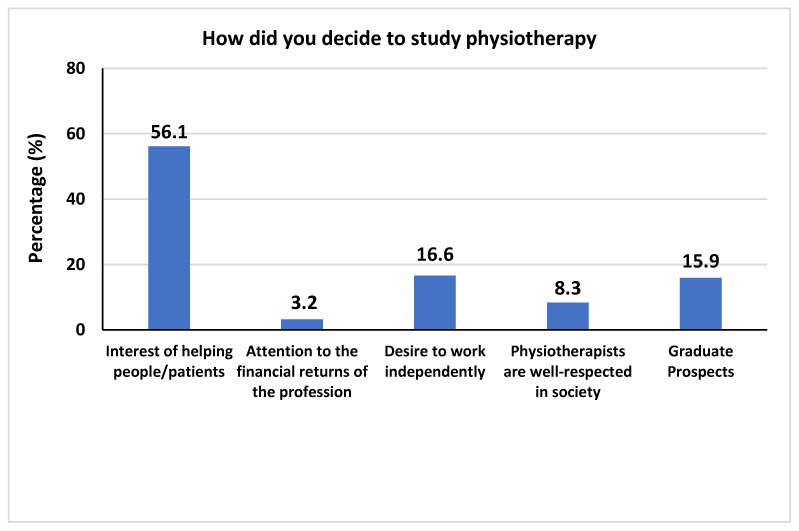
The frequencies and percentages of how students decided to study physiotherapy.

Figure 1 shows the question “How did you decide to study physiotherapy?” with the highest-percentage answer being “interest in helping people/patients” (56.1%), followed by “desire to work independently” in second place, with a percentage of 16.6%; followed by “graduate prospects”, with a percentage of 15.9%; then “physiotherapists are well-respected in society”, with a percentage of 8.3%; finally followed by “Attention to the financial returns of the profession”, with a percentage of 3.2%.

**Figure 2:**
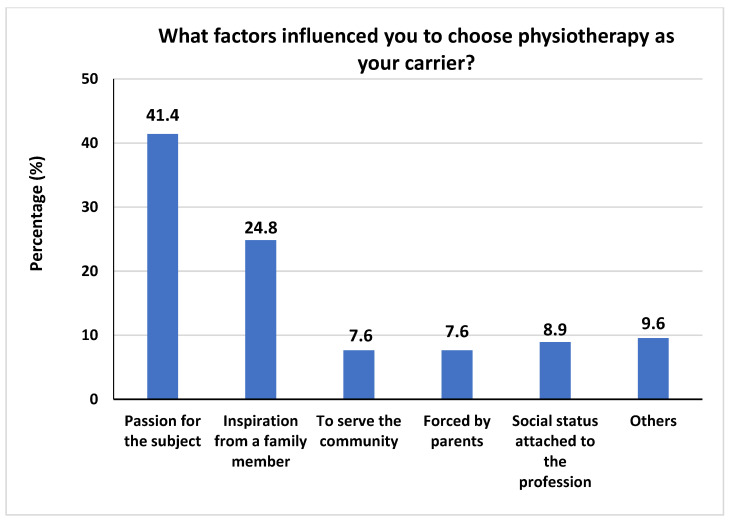
The frequencies and percentages of what factors influenced students to choose physiotherapy as your career.

On the other hand, Figure 2 illustrates the question “What factors influenced you to choose physiotherapy as your career?”. “Passion for the subject” received the highest percentage (35.7%), followed by “inspiration from a family member” (17.8%), “social status attached to the profession” (7.6%), “to serve the community”, and “forced by parents”, with a percentage (7.0%).

**Figure 3:**
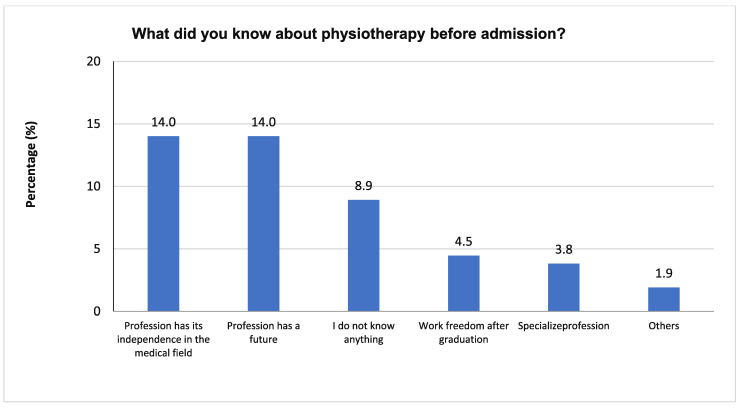
The frequencies and percentages of what students knew about physiotherapy before admission.

Figure 3 illustrates the question “What did you know about physiotherapy before admission?”. “Medical therapeutic profession” scored the highest percentage (52.9%), followed by “profession has its independence in the medical field” and “profession has a future” in second place (14.0%), “I do not know anything” in third place (8.9%), “work freedom after graduation” in fourth place (4.5%), and “specialize profession” in fifth place (4.5%). Then, there was a small percentage (3.8%) for “specialized occupation”, followed by an equal percentage (3.8%) for “others” (1.9%).

Table 2 displays the means and standard deviations. The participants’ estimates of the statements of the dimension “What kind of service does the physiotherapist offer to the patient” ranged between (3.45–4.27) with a high evaluation score, and statement (5) came first, which states, “Instructions on the home exercise program have a mean of (4.27) with a high degree”. Treatment intervention (1) is ranked last, which states “Treatment interventions” with a mean of 3.45 with a middle degree, and the mean of the dimension is 3.80 with a high evaluation score.

**Figure 4:**
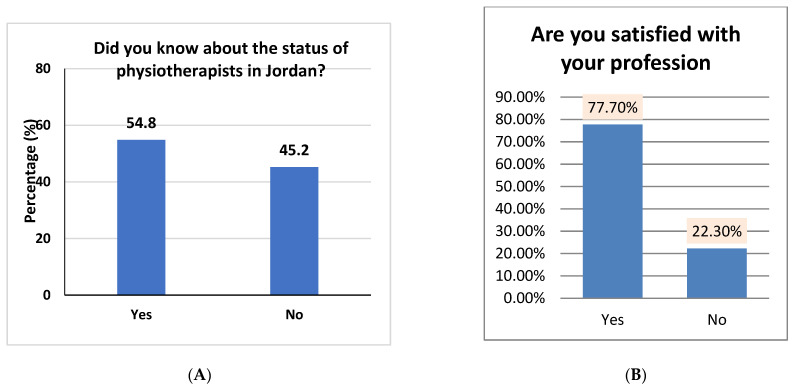
The frequencies and percentages for (**A**,**B**) questions.

Figure 4A depicts the results of these questions (A and B). “Did you know about the status of physiotherapists in Jordan?” indicates that the highest percentage (54.8%) was reached for the study participants who answered “Yes,” while the lowest percentage was (45.2%) for the study participants who answered “No.” (Figure 4A). However, the results of the survey question “Are you satisfied with your profession?” indicate that the highest percentage was reached (77.7%) for the study participants who answered “Yes,” while the lowest percentage was (22.3%) for the study participants who answered “No” (Figure 4B).

Table 3 shows the question “What kinds of opportunities do you think are available to develop your skills?”, with the highest percentage being “education and training” (49.0%); then “ability to make decisions and solve problems” in second place, with a percentage of 17.8%; followed by “teamwork”, with a percentage of 11.5%; “ability to plan and prioritize work”, with a percentage of 9.6%; “flexibility/adaptability”, with a percentage (5.1%); “ability to accept and learn from criticism”, with a percentage (3.8%); followed by “others” with a percentage (1.3%).

**Figure 5:**
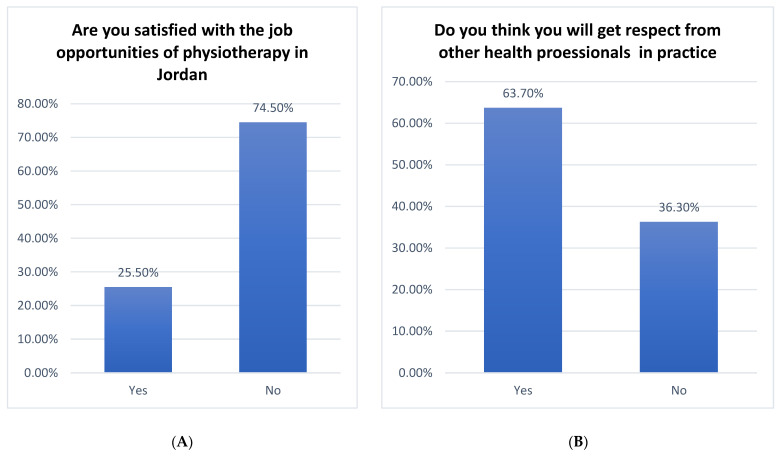
The frequencies and percentages for (**A**,**B**) questions.

In Figure 5A, the findings indicate that the highest percentage was reached (74.5%) for the study participants who answered “No” to “Are you satisfied with the job opportunities in Jordan?”, while the lowest percentage was (25.5%) for the study participants who answered “Yes” to “Are you satisfied with the job opportunities in Jordan?”. On the other hand, the findings of the question “Do you think you will get respect from other health professionals in practice?” revealed that the highest percentage was reached (63.7%) for the study participants who answered “Yes”, while the lowest percentage was (36.3%) for the study participants who answered “No” (Figure 5B).

**Figure 6:**
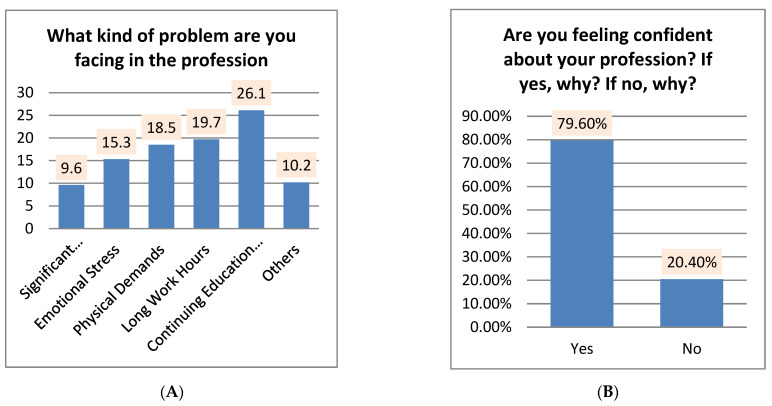
The frequencies and percentages for (**A**,**B**) questions.

Figure 6A illustrates the results of the question “What kind of problems are you facing in the profession?”, with the highest percentage being “continuing education requirements” 26.1%; “long work hours” in second place with a percentage of 19.7%; followed by “physical demands”, with a percentage of 18.5%; “emotional stress”, with a percentage of 15.5%; “others”, with a percentage of 10.2%; followed by “significant educational investment”, with a percentage of 9.6.

In Figure 6B, the findings of the question “Are you feeling confident about your profession? If yes, why? If no, why?” revealed that the highest percentage was reached 79.6% for the study participants who answered “Yes”, while the lowest percentage was 20.4% for the study participants who answered “No”.

Table 4 shows the means and standard deviation of the participants’ estimates of the statements in the dimension “How do you perceive your choice of PT as a career?”, ranging between 2.90–4.10, with a high evaluation score, and statements (5) and (4) were also highly evaluated. It states that “In general, the physical therapy profession’s career status is moving to a better status” and “In general, I am happy to be a physical therapy student”, with a mean of 4.10 and a high degree. The last statement (1) is ranked last, which states “I am satisfied with the current community image of the PT profession”, with a mean of 2.90 with a middle degree, and the mean of the dimension is 3.59, with a middle evaluation score.

## 4. Discussion

According to the findings of this study, all students answered the questions. At Jordan University of Science and Technology, many of the study participants were female (JUST). However, according to Sharma [17], most of the study participants were in the second year, with the lowest amount being in the fourth year. Furthermore, Jordanians account for the majority of the study’s participants, while Americans, Germans, Israelis, and Syrians compensate the minority. The majority of the participants in the study learned about physiotherapy from their families, while a smaller group learned about it from social media.

According to the findings, the factors that influenced respondents to choose physical therapy were the respondents’ passion for the subject, which was confirmed by Fathieh et al. [21], followed by the inspiration of family members, and finally the coercion of parents. The respondents’ familiarity with the profession of physical therapy, its independence in the medical field, and its future were attractive aspects. Furthermore, the type of service that the physiotherapist provides to the patient is also about instructions for the home exercise program, and most of the respondents were aware of the status of the physiotherapist in Jordan and are satisfied with the profession. This result is consistent with Turner [2], as he stated that the status and character of the profession depend greatly on the common interest of its members.

The results confirmed that education and training are two of the most important methods of developing skills and the ability to make decisions and solve problems. Whereas job opportunities for physiotherapy were available to older people, and they received approval and respect from other health professionals in practice and were highly trusted, these findings agree with the results of the studies of Richardson et al. [12], Lindquist et al. [5], and Sim [10]. In addition, the profession of physical therapy is moving into one of the best career positions. This result corresponds with conclusions from Lindquist [5]. Long working hours are one of the most severe problems facing the physiotherapy profession, along with continuous education needs. It is ideal for an individual to choose a profession that is in line with their own interests and skills. Today, it is noticed that individuals’ fears about the future prevent them from looking forward with confidence and affect their choice of profession. They are not satisfied with the disciplines they entered, and their dissatisfaction continues after graduation [22]. In a study looking at factors affecting university and career choices for students of the College of Health Sciences, it was observed that only 24% of students in the Department of Physical Therapy and Rehabilitation would make the same choice again if students were able to take the exam again [23]. In a study by Ohman et al. [24] conducted in Canada about the career choices and professional preferences of a group of Canadian physiotherapy students studying Physical Therapy and Rehabilitation, the reason students choose this career was stated as an opportunity to find a better job and the possibility of earning a higher salary [24]. Kunduracılar et al. stated in their study that physiotherapy students prefer this department mostly because of the possibility of finding a job and the students’ field of interest [25]. When Gotlib et al. investigated the attitudes of European physiotherapy students towards their chosen jobs, it was found that an interest in physical therapy was the main reason for choosing the Department of Physiotherapy [26]. Likewise, in this paper, it is evident that students willingly preferred this selection because it was so interesting. The interest in helping people or patients was the biggest reason for studying physiotherapy and also the desire, followed by interest in the profession, for income. Additionally, there was no difference in occupational awareness between those who voluntarily chose this career path and those who did not. However, there may be three main reasons why there is no difference between the two groups. The first is that both groups take occupational awareness courses; second, some of the students who registered, although they did not want to change their department, may choose again; and third, the remaining interest in their departments increased in subsequent years.

The participants for this paper consisted of students at the Jordan University of Science and Technology, as studies in the literature in this field contain a larger sample size of participants. In addition, in terms of the multi-center evaluation of the professional awareness of physiotherapy students in Jordan, it is the only work done in this field. Studies have failed to assess whether students who are enrolling in physical therapy despite being unwilling to enroll in it have increased their interest in the profession in the following years as well as the effects of professional independence, responsibility, strong university education, and social views on professional awareness. It can be argued that senior students have a good level of professional awareness. However, it is noted that some levels of occupational awareness are intermediate. However, it would be beneficial to organize activities such as seminars and interviews in order to increase the level of professional awareness. The researchers believe that such a prospective study could be undertaken in a broader sense, keeping in mind the limitations we mentioned above.

The current study was limited to a small participant size. Further studies should be done in the future that include larger participant sizes. In addition, other universities in the kingdom of Jordan should be included to compare the perceptions of the students at different cities in the kingdom. As people’s attitudes change with time, a comparison between the first- and last-year students’ perceptions should be made.

## 5. Conclusions

Physiotherapy education in Jordan is rising rapidly. The public, patients, parents, and clinical preceptors in physiotherapy settings must recognize this educational and professional practice. As a result, people should be more educated about physiotherapy. In Jordan, physiotherapy should be included in the health policy of Jordan. The authors recommend more research and studies on different areas in order to familiarize the public with the physiotherapy profession and build an appropriate marketing plan. Furthermore, the findings of the current study revealed that one of the most effective strategies for improving competence is education and training, followed by the ability to make decisions and solve problems. The ability to support people was the primary motivation for students studying physical therapy.

## Figures and Tables

**Table 1:** Shows the demographics characteristics of the samples (*n* = 157).

Demographic Characteristics		N (%)
Age	18 years	15 (9.8%)
	19 years	37 (23.6%)
	20 years	37 (23.6%)
	21 years	34 (21.7%)
	22 years	20 (12.7%)
	23 years	6 (3.8%)
	24 years	5 (3.2%)
	25 years	1 (0.6%)
	26 years	1 (0.6%)
	28 years	1 (0.6%)
Gender	Male	68 (43.31%)
	Female	89 (56.69%)
Nationality	Jordanian	129 (82.2%)
	Palestinian	4 (2.5%)
	Syrian	2 (1.3%)
	Nigerian	6 (3.2%)
	Egyptian	2 (1.3%)
	Kiwanian	2 (1.3%)
	Bahamian	5 (3.2%)
	American	1 (0.6%)
	German	1 (0.6%)
	Israel	1 (0.6%)
	Sudan	1 (0.6%)
Year levels	First year	33 (21%)
	Second year	55 (36.9)
	Third year	47 (29.9%
	Fourth year	18 (12.1%)
Where did you learn about physiotherapy?	Friends	39 (24.8%)
	Family	67 (42.7%)
	Daily news	34 (21.7%)
	Social media	17 (10.8%)

**Table 2:** Means and Standard Deviation (SD) for “What kind of service does the physiotherapist offer to the patient?”.

Statement	Frequency	Mean	SD	Rank	Level
Instructions on the home exercise program	5	4.27	0.94	1	High
Listening to patients’ concerns	4	3.89	0.98	2	High
Answering patients’ questions	3	3.82	0.96	3	High
Giving medical advice	2	3.55	1.08	4	Middle
Treatment interventions	1	3.45	1.31	5	Middle
Overall		3.80	0.83	-	High

**Table 3:** The frequencies and percentages of what kinds of opportunities students think are available to develop their skills.

Items	Frequency	Percentage
Education and training	77	49.0%
Teamwork	18	11.5%
Flexibility/adaptability	8	5.1%
Ability to make decisions and solve problems	28	17.8%
Ability to plan and priorities work	15	9.6%
Leadership/management skills	3	1.9%
Ability to accept and learn from criticism	6	3.8%
Others	2	1.3%
Total	157	100.0%

**Table 4:** Means and Standard Deviation (SD) for “How do you perceive your choice of PT as a career?”.

Statement	Frequency	Mean	SD	Rank	Level
In the general, the physical therapy profession/career status is moving to a better status	5	4.10	1.05	1	High
In general, I am happy to be a physical therapy student	4	4.10	1.05	1	High
I have no difficulty in obtaining my required continuing education requirements	3	3.78	1.03	3	High
The communication with PT professional organizations is unlimited	2	3.07	1.07	4	Middle
I am satisfied with the current community image of the PT profession	1	2.90	0.95	5	Middle
Overall		3.59	0.75	-	High

## Data Availability

The datasets used and analyzed during the current study are available from the corresponding author on reasonable request.
